# Genotyping of methicillin resistant *Staphylococcus aureus* from the United Arab Emirates

**DOI:** 10.1038/s41598-020-75565-w

**Published:** 2020-10-29

**Authors:** Abiola Senok, Rania Nassar, Handan Celiloglu, Anju Nabi, Mubarak Alfaresi, Stefan Weber, Irfan Rizvi, Elke Müller, Annett Reissig, Darius Gawlik, Stefan Monecke, Ralf Ehricht

**Affiliations:** 1 College of Medicine, Mohammed Bin Rashid University of Medicine and Health Sciences, P.O. Box 505055, Dubai, United Arab Emirates; 2 Oral and Biomedical Sciences, School of Dentistry, Cardiff University, Cardiff, UK; 3 Microbiology Department, Mediclinic City Hospital, Dubai Healthcare City, Dubai, United Arab Emirates; 4 Microbiology & Infection Control Unit, Pathology Department, Rashid Hospital, Dubai Health Authority, Dubai, United Arab Emirates; 5 College of Medicine, University of Sharjah, Sharjah, United Arab Emirates; 6 Reference Laboratory for Infectious Diseases, Shaikh Khalifa Medical City, Abu Dhabi, United Arab Emirates; 7 Leibniz Institute of Photonic Technology (IPHT), Jena, Germany; 8 InfectoGnostics Research Campus Jena, Jena, Germany; 9 PTC - Phage Technology Center GmbH, Bönen, Germany; 10 Institute of Infectious Diseases and Infection Control, University Hospital, Jena, Germany; 11 Institute for Medical Microbiology and Hygiene, Medical Faculty “Carl Gustav Carus”, Technische Universität Dresden, Dresden, Germany; 12 Institute of Physical Chemistry, Friedrich Schiller University Jena, Helmholtzweg 4, 07743 Jena, Germany

**Keywords:** Microbiology, Microbial genetics

## Abstract

Reports from Arabian Gulf countries have demonstrated emergence of novel methicillin resistant *Staphylococcus aureus* (MRSA) strains. To address the lack of data from the United Arab Emirates (UAE), genetic characterisation of MRSA identified between December 2017 and August 2019 was conducted using DNA microarray-based assays. The 625 MRSA isolates studied were grouped into 23 clonal complexes (CCs) and assigned to 103 strains. CC5, CC6, CC22 and CC30 represented 54.2% (n/N = 339/625) of isolates with other common CCs being CC1, CC8, CC772, CC361, CC80, CC88. Emergence of CC398 MRSA, CC5-MRSA-IV Sri Lanka Clone and ST5/ST225-MRSA-II, Rhine-Hesse EMRSA/New York-Japan Clone in our setting was detected. Variants of pandemic CC8-MRSA-[IVa + ACME I] (PVL+) USA300 were detected and majority of CC772 strains were CC772-MRSA-V (PVL+), “Bengal- Bay Clone”. Novel MRSA strains identified include CC5-MRSA-V (*edinA*+), CC5-MRSA-[VT + *fusC*], CC5-MRSA-IVa (*tst1*+), CC5-MRSA-[V/VT + *cas* + *fusC* + *ccrA/B-1*], CC8-MRSA-V/VT, CC22-MRSA-[IV + *fusC* + *ccrAA/(C*)], CC45-MRSA-[IV + *fusC* + *tir*], CC80-MRSA-IVa, CC121-MRSA-V/VT, CC152-MRSA-[V + *fusC*] (PVL+). Although several strains harboured SCC-borne fusidic acid resistance (*fusC*) (n = 181), erythromycin/clindamycin resistance (*ermC*) (n = 132) and gentamicin resistance (*aacA-aphD*) (n = 179) genes, none harboured vancomycin resistance genes while mupirocin resistance gene *mupR* (n = 2) and *cfr* gene (n = 1) were rare. An extensive MRSA repertoire including CCs previously unreported in the region and novel strains which probably arose locally suggest an evolving MRSA landscape.

## Introduction

Methicillin resistant *Staphylococcus aureus* (MRSA) is an important cause of nosocomial infections worldwide and is associated with significant patient morbidity, mortality and healthcare costs. The epidemiology of MRSA has been dynamic and with an evolution towards increasing predominance of community associated MRSA lineages (CA-MRSA) as agents of hospital acquired MRSA (HA-MRSA) infections^1,2^. In recent years, emerging data on the molecular characterisation of *S. aureus* isolates in the hospital and community setting in countries of the Arabian Gulf region has contributed to changing our understanding of the diversity of isolates present^3–9^. In accordance with reports from other parts of the world, community associated MRSA (CA-MRSA) lineages have overtaken HA-MRSA lineages as aetiological agents also of nosocomial infections. Interestingly, MRSA isolates circulating in the region represent a wide clonal diversity^3,8,10–12^. Furthermore, emergence of novel and variant MRSA strains including those with novel SCC*mec* elements or with complex elements that include *mecA* as well as a gene associated with fusidic acid resistance (*fusC*) continue to be reported^3,8,10^.

Although MRSA contributes to the burden of *S. aureus* infections in the United Arab Emirates (UAE), there remains a paucity of data on the molecular characterization of circulating strains. The UAE is a global tourist hub and home to a large expatriate population including those from regions from where there are hardly any data on MRSA prevalence and population structure. With this dynamic population, it is plausible that a diversity of MRSA clones can be found that includes highly transmissible pandemic clones as well as local strains from different regions from which tourists and expatriates might come to the UAE. Therefore, this study was carried out to determine the genotypes of MRSA isolates in the UAE and to differentiate closely related strains using a high-resolution typing method.

## Results

### CC diversity, virulence and antibiotic resistance genes

A total of 625 isolates obtained from wound/pus swabs (n = 459), blood cultures (n = 95), respiratory sites (endotracheal aspirates/sputum) (n = 50) and urine (n = 21) were included in the study. Based on DNA microarray analysis, these 625 MRSA isolates were grouped into 23 clonal complexes (CCs) which were assigned to 102 strains (Table 1) with “strains” being defined based on their CC affiliation, toxin gene carriage and SCC*mec* type (see Supplementary Table S1 online for details of strain assignment). The CCs were identified across study sites and there was no clustering observed of any specific CC based on study site or type of clinical specimen. Four predominant CCs accounted for 54.2% (n/N = 339/625) *of isolates.* These were CC30 (n = 91; 14.6%), CC5 (n = 89; 14.2%), CC22 (n = 82; 13.1%) and CC6 (n = 77; 12.3%) *(*Table 1*).* Other commonly identified CCs were CC1 (n = 48), CC8 (n = 40), CC772 (n = 36), CC361 (n = 35), CC80 (n = 27) and CC88 (n = 19). A majority of the strains belonged to CA-MRSA lineages harbouring SCC*mec* types IV and V (n/N = 610/625; 97.6%), (Supplementary Table S1 online). There was concordance between MRSA phenotypic and genotypic identification (data not shown).

**Table 1:** Clonal complex and strain assignments.

Clonal complex (CC) (# of isolates in CC)	Number of strain assignments
CC1 (n = 48)	7
CC5 (n = 89)	19
CC6 (n = 77)	3
CC8 (n = 40)	12
CC8/ST72 (n = 7)	2
CC9 (n = 1)	1
CC15 (n = 8)	1
CC22 (n = 82)	7
CC30 (n = 91)	6
CC45 (n = 3)	3
CC59 (n = 5)	2
CC80 (n = 27)	4
CC88 (n = 19)	6
CC96 (n = 1)	1
CC97 (n = 22)	4
CC121 (n = 8)	3
CC152 (n = 7)	3
CC239 (n = 11)	4
CC361 (n = 35)	5
CC398 (n = 3)	2
CC772 (n = 36)	4
CC1153 (n = 4)	2
CC2250 *S. argenteus* (n = 1)	1

We detected isolates harbouring SCC-borne fusidic acid resistance (*fusC*) gene (n = 181), erythromycin/clindamycin resistance *(ermC)* gene (n = 132), and bifunctional enzyme gentamicin resistance (*aacA-aphD*) (n = 179). In addition, 25 isolates carried the fusidic acid resistance gene *fusB* (also known as *far1*). Carriage of the mupirocin resistance gene *mupR* (n = 2) and 23S rRNA methyltransferase *cfr* gene (n = 1) was rare while none of our strains carried vancomycin / teicoplanin resistance genes (*vanA*, *vanB, vanZ*). The Panton Valentine leukocidin (*pvl*) genes were present in 49% of all isolates studied while 13.8% were positive for the toxic shock syndrome (*tst-1*) gene. The prevalence rates of the antibiotic resistance and virulence genes are shown in Tables 2 and Table 3 respectively.

**Table 2:** Detection of antibiotic resistance gene.

Antibiotic resistance genes		# Positive (N = 625)	% positive
Alternate penicillin binding protein 2, defining MRSA	*mecA*	625	100.0
Mercury resistance operon	*merA; merB*	0	0.0
SCC*mec* XI	*mecC; blaZ-SCCmec XI*	0	0.0
Beta-lactamase operon	*blaZ ; blaI; blaR*	588	94.1
rRNA adenine N-6-methyl-transferase, erythromycin/clindamycin resistance	*ermA*	15	2.4
Erythromycin/clindamycin resistance	*ermB*	4	0.6
Erythromycin/clindamycin resistance	*ermC*	132	21.1
Lincosamide Nucleotidyltransferase	*linA*	10	1.6
Energy-dependent efflux of erythromycin	*msrA*	101	16.2
Acetyl-transferase inactivating streptogramin A	*vatB*	0	0.0
ATP binding protein, Streptogramin A resistance	*vgaA*	0	0.0
Bifunctional enzyme gentamicin resistance	*aacA-aphD*	179	28.6
Amino-glycoside adenyl-transferase, tobramycin resistance	*aadD*	47	7.5
3′5′-Aminoglycoside phosphotransferase, neo-/kanamycin resistance	*aphA3*	138	22.1
Streptothricin acetyltransferase	*sat*	129	20.6
Dihydrofolate reductase type 1	*dfrA*	87	13.9
Fusidic acid resistance	*fusB*	25	4.0
Hypothetical protein associated with fusidic acid resistance	*Q6GD50 (fusC)*	181	29.0
Mupirocin resistance protein	*mupR*	2	0.3
Tetracycline resistance	*tetK*	85	13.6
Tetracycline resistance	*tetM*	18	2.9
Chloramphenicol acetyltransferase	*cat*	5	0.8
23S rRNA methyltransferase	*cfr*	1	0.2
Chloramphenicol/florfenicol exporter	*fexA*	10	1.6
Metallothiol transferase	*fosB*	403	64.5
	*fosB-plasmid*	0	0.0
Quaternary ammonium compound resistance protein A/B	*qacA; qacC*	6	1.0
Transport-/efflux protein	*tetEfflux*	539	86.2
Vancomycin resistance genes	*vanA; vanB*	0	0.0
Teicoplanin resistance gene from enterococci	*vanZ*	0	0.0

**Table 3:** Detection of virulence genes.

Virulence genes		# Positive (N = 625)	% positive
Toxic shock syndrome toxin 1	*tst1*	86	13.8
Panton Valentine leukocidin F/S component	*lukF-PV; lukS-PV*	306	49.0
Staphylokinase	*sak*	557	89.1
Chemotaxis-inhibiting protein	*chp*	272	43.5
Staphylococcal. Complement inhibitor	*scn*	597	95.5
Exfoliative toxin serotype A	*etA*	3	0.5
Exfoliative toxin serotype B	*etB*	1	0.2
Exfoliative toxin D	*etD*	27	4.3
Epidermal cell differentiation inhibitor A	*edinA*	9	1.4
Epidermal cell differentiation inhibitor B	*edinB*	32	5.1
Epidermal cell differentiation inhibitor C	*edinC*	1	0.2
Arginine catabolic mobile element locus	*ACME*	17	2.7
*Staphylococcus aureus* surface protein G	*sasG*	512	81.9

### Emerging clonal complexes

Our findings document the first identification of CC398 MRSA in the Arabian Gulf region. The detection of *pvl* genes and SCC*mec* typing using a second array revealed it to belong to the Asian, human adapted lineage rather than to the European livestock-associated one. Among CC5, we report the emergence of CC5-MRSA-IV Sri Lanka Clone and a second identification of ST5/ST225-MRSA-II, Rhine-Hesse EMRSA/New York-Japan Clone in the region (Supplementary Table S1 online). The CC5-MRSA-IV, Sri Lanka Clone is a CC5-MRSA carrying a SCC*mec* IVc element and *pvl* genes as well as, variably, the enterotoxin genes *sed, sej* and *ser*. In CC8, different variants of the pandemic CC8-MRSA-[IVa + ACME I] (PVL+) USA300 strain were detected including one that lost *arc* genes and *speG* while retaining *opp* and *copA2-*SCC (copper resistance), as well as putative PVL-deletion mutants (Supplementary Table S1 online). For the first time in our region, we report identification of the ACME-negative/PVL-positive CC8-MRSA-[SCC*mec* IVc + Hg], which is a strain frequently described from Spain and Latin America (Supplementary Table S1 online). Another emerging strain was CC22-MRSA-IV harbouring *pvl* and *tst1* genes. A majority of the CC772 MRSA was assigned to CC772-MRSA-V (PVL+), "Bengal Bay Clone" while some of the CC772 isolates presented with other, unusual SCC*mec* variants.

### Novel variant MRSA strains

We identified novel variant MRSA strains from 9 CCs, namely:

CC5: Four novel variant strains were identified, three of which carried SCC*mec* type V. All the strains carried regulatory and capsular genes *agrII* and *cap5* respectively. Although all harboured the *egc cluster* enterotoxin genes, the *edinA and tst-1* genes were found in single strains respectively. The CC5-MRSA-IVa (*tst1*+) was the only one with SCC*mec* type IV and it uniquely harboured SCC*mec* IVa instead of usual IVc. Both CC5-MRSA-[VT + *fusC*] and CC5-MRSA-[V/VT + *cas* + *fusC* + *ccrA/B-1*] carried the *fusC* gene. The CC5-MRSA-[VT + *fusC*] with SCCmec VT + *fusC* was identified as an unknown CC5/72 strain. A novel SCC*mec* element was exhibited by CC5-MRSA-[V/VT + *cas* + *fusC* + *ccrA/B-1*] which also carried the highest repertoire of antibiotic resistance genes among the novel strains (Table 3).
CC8: The novel variant strain in this CC was the CC8-MRSA-V/VT which uniquely harboured a SCC*mec* V sub-type (as in WIS; GenBank AB121219.1) and harboured the cassette chromosome recombinase genes *ccrC* on the SCC*mec* element. No toxin associated genes were identified in this strain.
CC22: The CC22-MRSA-[IV + *fusC* + *ccrAA/(C)*] had a new SCC*mec* element characterised by carriage of SCC*mec* type IV with *ccrAA/C* recombinase genes and *fusC* gene. In addition to *egc* cluster which is usually found in CC22 MRSA, this strain also harboured *tst1* gene.
CC45: The CC45-MRSA-[IV + *fusC* + *tir*] harboured the combination of *fusC* and *tirS* genes on the SCC*mec* element making is a novel CC45 variant strain.
CC80: CC80-MRSA nearly always harbour PVL and SCC*mec* IVc while lacking enterotoxin genes. However, this novel variant had SCC*mec* IVa, harboured enterotoxin genes (*seb, sek, seq*) and was negative for *pvl* genes.
CC121: Subtyping of the CC121 strains revealed the presence of a variant CC121-MRSA- VT strain with the SCC*mec* VT (GR1).
CC152: The CC152-MRSA-[V + *fusC*] (PVL+) is the only novel variant strain harbouring the *pvl* genes. The carriage of SCC [*mec* V + *fusC*] is novel in this CC.
CC361: One CC361 strain uniquely carried an SCC*mec* V / *cas* composite element. It also harboured *tst1* gene and a complement of enterotoxin genes (*sec, sel, egc cluster)*.
CC1153: Two PVL-positive CC1153-MRSA isolates were observed that presented with a *mec* complex B, *ccrA/B1* recombinase genes and the *fusC* gene. Table 4 shows the genetic characterization of the novel strains.

**Table 4:** Characterization of novel methicillin-resistant *Staphylococcus aureus* strains.

Clonal complex	Novel variants	SCC *mec*-complex associated genes	Regulatory and capsule genes	Antibiotic resistance genes	Toxin associated virulence genes	Other virulence genes
CC5	CC5-MRSA-V (*edinA*+) (n = 1)	*ugpQ; mecA; ccrA-2; ccrB-2*	*agrII; cap5*	*blaZ; blaI; blaR; fosB; tetEfflux*	*egc* cluster	*hla; sak; chp; scn; edinA; icaA/C/D; clfA/B; fnbA/B*
	CC5-MRSA-[VT + *fusC*] (n = 2)	*ugpQ; mecA;* Q6GD50 *(fusC); ccrAA; ccrC*	*agrII; cap5*	*blaZ; blaI; blaR; fosB; tetEfflux*	*seb; egc* cluster	*hla; sak; scn; icaA/C/D; clfA/B; fnbA/B*
	CC5-MRSA-IVa (*tst1*+) (n = 1)	*ugpQ; mecA; ccrA-2; ccrB-2*	*agrII; cap5*	*blaZ; blaI; blaR; fosB; tetEfflux*	*tst1; sea; egc* cluster	*hla; sak; chp; scn; icaA/C/D; clfA/B; fnbA/B*
	CC5-MRSA-[V/VT + *cas* + *fusC* + *ccrA/B-1*] (n = 1)	*ugpQ; mecA; ccrA-1; ccrB-1; ccAA; ccrC;* Q6GD50 *(fusC); cas*	*agrII; cap5*	*blaZ; blaI; blaR; ermC; tetK; tetM; cat; fexA; fosB; tetEfflux*	*sea; sed; sej; ser; egc* cluster	*hla; sak; scn; icaA/C/D; clfA/B*
CC8	CC8-MRSA-V/VT (n = 1	*ugpQ; mecA; ccrC*	*agrI; cap5*	*blaZ; blaI; blaR; far1; fosB; tetEfflux*	*–*	*hla; icaA/C/D; clfA/B; fnbA*
CC22	CC22-MRSA-[IV + *fusC* + *ccrAA/(C*)] (n = 1)	*ugpQ; mecA; ccAA; ccrC;* Q6GD50 *(fusC)*	*agrI; cap5*	*blaZ; blaI; blaR*	*tst1; egc* cluster	*hla; sak; chp; scn; icaA/C/D; clfA/B; cna; fnbA/B*
CC45	CC45-MRSA-[IV + *fusC* + *tir*] (n = 1)	*ugpQ; mecA; ccrA-2; ccrB-2;* Q6GD50 *(fusC); tirS*	*agrI; cap8*	*tetEfflux*	*sek; egc* cluster	*hla; sak; chp; scn; icaA/C/D; clfA/B; fnbA*
CC80	CC80-MRSA-IVa (n = 1)	*ugpQ; mecA; ccrA-2; ccrB-2*	*agrIII; cap8*	*blaZ; blaI; blaR; ermC; tetEfflux*	*seb; sek; seq;*	*hla; sak; chp; scn; etD; edinB; icaA/C/D; clfA/B; fnbA/B*
CC121	CC121-MRSA-V/VT (n = 1)	*ugpQ; mecA; ccrAA; ccrC;* Q6GD50 *(fusC)*	*agrIV; cap8*	*blaZ; blaI; blaR; aacA-aphD; fosB; tetEfflux*	*egc* cluster	*hla; sak; scn; etA; etB; edinC; icaA/C/D; clfA/B; fnbA/B*
CC152	CC152-MRSA-[V + *fusC*] (PVL+) (n = 1)	*ugpQ; mecA; ccrAA; ccrC; Q6GD50 (fusC)*	*agrI; agrIV; cap5*	*blaZ; blaI; blaR; ermC; aacA-aphD; tetK; tetEfflux*	*lukF-PV; lukS-PV*	*hla; sak; scn; edinB; icaA/D; clfA/B; fnbA/B*
CC361	CC361-MRSA-V (SCC*mec* V/*cas* composite element) (n = 2)	*ugpQ; mecA; ccrAA; ccrC;*	*agrI; cap8*	*blaZ; blaI; blaR; ermC; aphA3; sat; tetK; fosB; tetEfflux*	*tst1; sec; sel; egc* cluster	*hla; sak; chp; scn; icaA/C/D; clfA/B; fnbA/B*
CC1153	CC1153-MRSA with SCC*me*c I + *fusC* (PVL+) (n = 2)	*ugpQ; mecA; ccrA-1; ccrB-1;* Q6GD5*0 (fusC)*	*agrII; cap5*	blaZ; blaI; blaR; ermC*; tetEfflux	*lukF-PV; lukS-PV*	*hla; sak; scn; icaA/C/D; clfA/B; fnbA/B*

*Present in one isolate; *agr* accessory gene regulator*; ccr* cassette chromosome recombinase gene*; ugpQ* glycerophosphoryl diester phosphodi-esterase, associated with mecA; *mecA* alternate penicillin binding protein 2*,* defining MRSA*; Q6GD50 (fusC)* hypothetical protein associated with fusidic acid resistance*; cap*, capsule gene; *blaZ* beta-lactamase; *blaI* beta lactamase repressor (inhibitor); *blaR* beta-lactamase regulatory protein*; fosB* Metallothiol transferase*; tetEfflux* transport-/efflux protein*; tetM/K* tetracycline resistance markers*; ermC;* rRNA adenine *N*-6-methyl-transferases causing erythromycin/clindamycin resistance*; cat* chloramphenicol acetyltransferase; *fexA* chloramphenicol/florfenicol exporter*; aacA-aphD* bifunctional enzyme gentamicin resistance; *aphA3* 3′5′-aminoglycoside phosphotransferase, neo-/kanamycin resistance*; sat* streptothricin acetyltransferase; *far1* fusidic acid resistance; *egc* cluster*:* enterotoxins g,i,m,n,o,u; *sea* enterotoxin A; *seb* enterotoxin B*; sec* enterotoxin C; *sed* enterotoxin D*; sej,* enterotoxin J*; sek* enterotoxin K*; sel* enterotoxin L*; seq* enterotoxin Q*; tst1* toxic shock syndrome toxin 1*; lukF-PV/lukS-PV,* Panton Valentine leukocidin F/S component*; hla*, haemolysin alpha; *sak* staphylokinase*; scn* staphylococcal complement inhibitor*; chp* chemotaxis-inhibiting protein (CHIPS); *edinA * intercellular adhesion protein A/C/D*; clfA/B* clumping factor A/B*; fnbA/B* fibronectin-binding protein A/B.

## Discussion

In recent years, emerging data globally and specifically in the Arabian Gulf region, have shown an evolving MRSA epidemiology with a shift to predominance of CA-MRSA lineages in nosocomial infections and an emergence of novel strains leading to an increased biological diversity of MRSA strains as well as to a greater diversity of SCC*mec* elements and variants thereof. In the UAE, MRSA contributes to the burden of infection and 30% of MRSA isolates identified at a tertiary care facility between 2011 and 2012 were CA-MRSA lineages^13^. Indeed, Sonnevend et al. also reported a trend of increasing CA-MRSA lineages between 2003 and 2008 at another tertiary care facility in the UAE^14^. However, data on the molecular characterization and strain assignments of circulating MRSA in the UAE are lacking. With the rapidly evolving changes reported in neighbouring countries and the dynamic population of the UAE, this study provides a much-needed snapshot of the genetic make-up of MRSA strains circulating in the UAE. Our findings demonstrate that an extensive MRSA repertoire of predominantly CA-MRSA lineage including CCs previously unreported in the region, plus rare and novel strains are present in the UAE. While the predominance of CC5, CC6, CC22 and CC30 is in accordance with reports from other countries in the region, identification of several pandemic MRSA strains and their variants such as CC8-MRSA-[IVa + ACME I] (PVL+), USA300, CC22-MRSA-IV UK-EMRSA-15/Barnim EMRSA; CC30-MRSA-IV (PVL+), Southwest Pacific Clone and the HA-MRSA lineage CC239-MRSA-[*mec* III + *Cd/Hg* + *ccrC*] is of concern in light of enhanced virulence, fitness and survivability of these strains. Furthermore, the first identification of CC398 MRSA heralds ominously the appearance of yet another previously unreported clonal complex in our region. The CC398 MRSA identified is the PVL-positive human variant of MRSA CC398 which is believed to have originated in South East Asia and is a frequent cause of infections in China and Vietnam^15–17^. In Europe, links to South East Asia were demonstrated in cases of infections and outbreaks associated with this CC398-MRSA strain^15,18^. It is therefore highly likely that this strain was introduced to the UAE from South East Asia or even possibly via Europe.

CC5-MRSA are globally common^19^ with CC5-MRSA-IV (PVL + */edinA*+), WA MRSA-121, CC5-MRSA-[IV + *fus* + *ccrAB*], “Maltese Clone” and CC5-MRSA-IV (*tst1*+), being the prevalent strains in the Arabian Gulf region^3,4,6,20^. Recently, the emergence of CC5-MRSA-VI strain including a novel variant which harboured the Staphylococcal TIR-protein binding protein gene (*tirS)* as additional payload on SCC*mec* was reported in Saudi Arabia ^8^. Identification of eight CC5-MRSA-[VI + *fusC*] including two with *tirS* in the current study suggests dissemination of this strain in the region. It has been postulated that the *tirS* gene confers enhanced bacterial survival and because it is located on a mobile genetic element in *S. aureus*, in this case an SCC element together with *fusC,* horizontal gene transfer among MRSA strains is likely^8,21^. In addition to the CC5-MRSA we also identified two other strains with *tirS* namely a novel CC45 variant, CC45-MRSA-[IV + *fusC* + *tir*], and CC1-MRSA [VT + *fusC* + *tir* + *ccrAB1*]. These findings are supportive of the notion for on-going horizontal gene transfer among MRSA strains in our setting.

The occurrence of antibiotic resistance and virulence genes on MRSA SCC*mec/fus* genetic elements is suggestive of novel adaptive mechanisms^22^. A high consumption of fusidic acid in the population confers a selective advantage for the emergence and proliferation of strains carrying the *fusC* gene^23^. When *fusC* and *mecA* co-exist on the SCC element, fusidic acid use could promote MRSA in the community while beta-lactam use promotes fusidic acid resistance in the hospital. Hence, MRSA strains with SCC*mec* + SCC*fus* composite elements have a selective advantage in both the hospital and community settings. The high prevalence of MRSA strains with *fusC* gene plus presence of CC80 MRSA (which usually harbour *fusB*) suggests on-going community misuse of fusidic acid as a driving factor for MRSA evolution in our setting. Therefore, future emergence of MRSA strains with increased bacterial fitness, resistance and virulence is possible, hence continued surveillance for early detection and responsible use of antibiotics is necessary.

Recently reported phylogenetic analysis of CC5 PVL-positive MRSA from four continents showed geographical clustering with the identification of the ST5-PVL-positive MRSA-IVc Sri Lanka clone^24^. Wider geographical spread of this clone was demonstrable with its identification in England and Australia where demonstrable links to Sri Lanka were not consistently established^24^. Our study documents the first report of CC5-MRSA-IV Sri Lanka Clone with two distinct variants based on carriage or absence of enterotoxin genes (*sed, sej, ser*) in our setting. The detection of CC5-MRSA-IV Sri Lanka Clone also underscores the need for continued surveillance to keep track of introduction of new MRSA strains into the population. While it is likely that this strain was introduced rather than arising de novo in the UAE, we were unable to establish direct travel links with Sri Lanka probably because this information was sought retrospectively and patients might have acquired the strains through contact with healthcare workers or carriers with epidemiological links. The ST5/ST225-MRSA-II, Rhine-Hesse EMRSA/New York-Japan Clone identified in this study is a HA-MRSA lineage pandemic strain which has been reported in Europe, Asia, North America and Australia^19,25^. It has previously been identified in Kuwait and our findings demonstrate the second report in our region. Although variants of this MRSA strain harbouring arginine catabolic mobile element (ACME) have been reported, this was not evident in the strain found in this study^25^. Further identification of novel strains within CC5 including *edinA* + variant, and strains with new SCC*mec* elements including SCC [VT + f*usC*] and SCC-[V/VT + *cas* + *fusC* + *ccrA/B-1*] are suggestive of ongoing genetic modification and recombination, among CC5-MRSA strains circulating in the UAE, or of importation from yet unidentified sources.

CC22 is a widespread clonal group which is prevalent in this region. Using high-resolution typing methods with SCC*mec* subtyping we had previously identified the regional presence of six distinct C22-MRSA-IV strains^11^ and recently an additional novel variant with SCC*mec* V/VT + *fusC* and *pvl* was reported from Saudi Arabia^8^. In this study, these previously reported strains as well as the European pandemic strain CC22-MRSA-IV (UK-EMRSA-15/Barnim EMRSA) were detected, indicative of strong links to Middle East, India and Europe for the evolution of CC22-MRSA in the UAE. In addition, our findings reflect on-going expansion of the diversity of CC22 MRSA strains with the identification of the previously undescribed CC22-MRSA-[IV + *fusC* + *ccrAA/(C*) which harboured a novel SCC*mec* element.

The CC772-MRSA-V (PVL+), Bengal Bay Clone is believed to have emerged in the Indian subcontinent from the same lineage as the widespread CC1 and CC5 strains ^26–28^. In a recent report from Pakistan, a majority of the characterised MRSA strains belonged to this CC^29^. In Western Europe, infections with CC772-MRSA are commonly associated with previous travel or patient´s ethnic origin from the Indian sub-continent ^26^. Having been previously reported in Saudi Arabia, Oman and Kuwait, its identification in the UAE is not surprising giving the dynamic population movement with the Indian subcontinent^4,9,30^.

MRSA CC121, CC152, CC361, and CC1153 are considered to be emerging in our region as variant strains belonging to these CCs are continually reported. It is therefore not surprising that other novel variant strains identified in this study belonged to these CCs ^3,8^. CC152-MRSA-V has been reported from Germany, Australia, Sweden, Switzerland, the Balkans, Saudi Arabia and Kuwait^3,8,19,31^. In accordance with reported literature, the CC152-MRSA-V identified in this study harbour the *pvl* and *edinB* genes, and lacked enterotoxin genes^19^. This strain carried erythromycin/clindamycin resistance *ermC* gene which was not present in previously described strains^3,8^.

The first identification of CC361 in Saudi Arabia was recently reported with detection of variant strains similar to those previously described in Kuwait indicative of regional spread^3,8^. Although CC361-MRSA had previously been described as a rare strain in the UAE^19^, and only one CC361-MRSA-[V/VT + *fusC*] was found as nasal colonizer in a recent study from UAE dental clinics^32^, the current findings suggest a wider occurrence of this CC. Similar to our findings, a recent report from Kuwait describing 102 novel MRSA variants identified 32 as CC361 strains^3^. With up to 5% (n/N = 35/625) of our isolates belonging to CC361-MRSA inclusive of a novel strain with SCC*mec* V / *cas* composite element, it appears that we can no longer consider this as a rare MRSA lineage in our setting.

In conclusion, an extensive MRSA repertoire which includes CCs previously unreported in the region, plus pandemic, rare and novel variant strains are present in the UAE. Some strains detected occur in other countries, so a travel connection is possible. Others which have not been described elsewhere, probably evolved within the region. The lack of documentation of travel history for our patients is a limitation of this study as such information would have been helpful in mapping the travel connections. However, in a population as diverse as the UAE, it should be noted that importation of a strain from abroad could have multiple sources and prior travel by the patient represents only one piece of the puzzle. An on-going, sustained transmission of at least some of the “imported” clones might have already started within the UAE population and affecting individuals who previously did not travel. Nevertheless, our findings provide the first detailed information on the genotyping of MRSA isolates in the UAE, providing important baseline data for future surveillance work and whole genome sequencing of novel strains to help understand MRSA evolution and epidemiology in the UAE.

## Materials and methods

Specimen collection and bacterial strains: MRSA isolates were identified between December 2017 and August 2019 at four diagnostic microbiology laboratories associated with secondary and tertiary care facilities, across three Emirates (Dubai, Abu Dhabi and Umm Al Quwain) in the UAE, with no routine inter-hospital transfer of patients. Apart from Abu Dhabi isolates which were obtained between December 2017 and August 2018, those from other Emirates were identified between December 2017 and August 2019. Only consecutive MRSA isolates associated with clinical infection were included with only one isolate per patient. There was no restriction on patient age for inclusion of isolates. However, all isolates obtained from specimens (nasal/axillary swabs) collected for MRSA screening were excluded. Identification of *S. aureus* and confirmation of methicillin resistance were performed using the Vitek automated platform (bioMérieux, Marcy-l'Étoile, France) in the diagnostic laboratory in accordance with manufacturer’s instructions and Clinical and Laboratory Standards Institute guidelines^33^. Ethical approval was obtained from Mohammed Bin Rashid University, Dubai Scientific Research and Mediclinic Hospitals ethics committees (MBRU-IRB-2018–019; DSREC-05/2018_11 and CR/2018/42). As the study involved use of MRSA isolates already identified as part of routine diagnostic investigation, waiver of informed consent was granted by the above-mentioned ethics committees. All methods in the study were carried out in accordance with relevant guidelines and regulations. All MRSA isolates were stored at -80 °C pending molecular characterisation.

Molecular characterisation of isolates was carried out using the StaphyType DNA microarray (Abbott [Alere Technologies GmbH], Jena, Germany). The previously described probes, primers, and procedures were used for the detection of species markers, virulence and resistance genes as well as for SCC*mec* subtyping^19,34,35^. Microarray images were taken and analysed using the dedicated reader and software (Alere Technologies). The analysis of presence or absence of target gene, assignment to clonal complex, sequence type and strains as well as SCC*mec* type was carried out as previously described^19,34,35^. Selected isolates were further characterised using a second array that facilitated assignment to SCC*mec* subtypes^35^.

## Supplementary information

Supplementary Information.

## Data Availability

Data generated or analysed during this study are included in this published article.
