# Supplementary material for: Genotyping of methicillin resistant Staphylococcus aureus from the United Arab Emirates
Source: Sci Rep. 2020 Oct 29;10:18551. doi: 10.1038/s41598-020-75565-w (PMC7596093; doi:10.1038/s41598-020-75565-w)
Supplement: Supplementary file 1 — Supplementary Information. [file 41598_2020_75565_MOESM1_ESM.docx]

**Genotyping of methicillin resistant *Staphylococcus aureus* from the United Arab Emirates**

Abiola Senok^1*^, Rania Nassar^1,2^, Handan Celiloglu^1,^^3^, Anju Nabi^4^, Mubarak Alfaresi^5^, Stefan Weber^6^, Irfan Rizvi^3^, Elke Müller^7,8^, Annett Reissig^7,8^, Darius Gawlik^9, 10^, Stefan Monecke^7,8,11^, Ralf Ehricht^7,8,12^

^1^College of Medicine, Mohammed Bin Rashid University of Medicine and Health Sciences, Dubai, United Arab Emirates

^2^Oral and Biomedical Sciences, School of Dentistry, Cardiff University, Cardiff, United Kingdom

^3^Microbiology Department, Mediclinic City Hospital, Dubai Healthcare City, Dubai, United Arab Emirates

^4^Microbiology & Infection Control Unit, Pathology Department, Rashid Hospital, Dubai Health Authority, Dubai, United Arab Emirates

^5^College of Medicine, University of Sharjah, Sharjah, United Arab Emirates

^6^Reference Laboratory for Infectious Diseases, Sheikh Khalifa Medical City, Abu Dhabi, United Arab Emirates

^7^Leibniz Institute of Photonic Technology (IPHT), Jena, Germany

^8^ InfectoGnostics Research Campus Jena, Jena, Germany

^9^PTC - Phage Technology Center GmbH, Bönen, Germany

^10^Institute of Infectious Diseases and Infection Control, University Hospital, Jena, Germany

^11^Institute for Medical Microbiology and Hygiene, Medical Faculty "Carl Gustav Carus", Technische Universität Dresden, Dresden, Germany

^12^Friedrich Schiller University Jena; Institute of Physical Chemistry; Helmholtzweg 4; 07743 Jena; Germany

**Supplementary Table S1: Assignment of study isolates into clonal Complexes and strains**

| **Clonal Complex** | **Strain assignment** | **Number of strains** |
| --- | --- | --- |
| CC1 (n=48) | CC1-MRSA-[V/VT+*fusC*] (PVL+) | 2 |
|  | CC1-MRSA-[IV+*fusC+ccrAB1*], WA MRSA-1/45 | 10 |
|  | CC1-MRSA-[V/VT+*fusC+ccrAB1*] | 17* |
|  | CC1-MRSA-[V/VT+*fusC+ccrAB1*] (PVL+) | 14 |
|  | CC1-MRSA-IV (*aphA3/sat*-positive) | 2 |
|  | CC1-MRSA-IV, WA MRSA-1/57 (*aphA3/sat-*negative) | 2 |
|  | CC1-MRSA-V/VT | 1 |
| CC5 (n=89) |  |  |
|  | CC5-MRSA-[V/VT+*fusC*] | 33 |
|  | CC5-MRSA-[V/VT+*fusC*] | 10** |
|  | CC5-MRSA-[VI+*fusC*] | 8*** |
|  | CC5-MRSA-IVc (*sed/j/r+,* PVL+), "Sri Lanka Clone" | 9 |
|  | CC5-MRSA-IVc *(sed/j/r-,* PVL+), "Sri Lanka Clone" | 5 |
|  | CC5-MRSA-[V/VT+*fusC*] (PVL+) | 5 |
|  | CC5-MRSA-IV *(tst1+*) | 4^@^ |
|  | CC5-MRSA-IV (*edinA+*) | 2 |
|  | CC5-MRSA-IV (PVL+/*edinA+*), WA MRSA-003/121 | 2 |
|  | CC5-MRSA-V/VT, WA MRSA-81/85/86/123 | 2 |
|  | CC5-MRSA-[I+*fusC*], Geraldine Clone | 1 |
|  | CC5-MRSA-[IV+*fusC+ccrAB*], Maltese Clone | 1 |
|  | CC5-MRSA-[V/VT+*fusC+ccrA/B-1*] (PVL+) | 1 |
|  | CC5-MRSA-IV (PVL+/*edinA+*), WA MRSA-121 | 1 |
|  | CC5-MRSA-IV (*sed/j/r+)* | 1 |
|  | CC5-MRSA-V (*edinA+*) | 1 |
|  | CC5-MRSA-V/VT (PVL+) | 1 |
|  | CC5-MRSA-V/VT *(sed/j/r+*), WA MRSA-11/34/35/87/90/108 | 1 |
|  | ST5/ST225-MRSA-II, Rhine-Hesse EMRSA/New York-Japan Clone | 1 |
| CC6 (n=77) |  |  |
|  | CC6-MRSA-IV, WA MRSA-51 | 63 |
|  | CC6-MRSA-IV | 9 |
|  | CC6-MRSA-IV (PVL+) | 5 |
| CC8 (n=40) |  |  |
|  | CC8-MRSA-[IVa+ACME I] (PVL+), USA300 | 14 |
|  | CC8-MRSA-IV (no enterotoxin genes) | 6 |
|  | CC8-MRSA-V/VT | 5^#^ |
|  | CC8-MRSA-[IV+ACME], putative PVL-deletion mutant of USA300 | 3 |
|  | CC8-MRSA-IVb/d/i (PVL+, *sed/j/k/q/r+*) | 3 |
|  | CC8-MRSA-[VI+*fusC*] | 2 |
|  | CC8-MRSA-IV (*tst1+*) | 2 |
|  | C8-MRSA-[IVc+Hg] (PVL+), ACME-negative/Spanish or Latin American variant of USA300 | 1 |
|  | CC8-MRSA-[IVa+truncated ACME] (PVL+), USA300 variant | 1 |
|  | CC8-MRSA-[V/VT+f*usC*] | 1 |
|  | CC8-MRSA-IV (*seb/k/q+*) | 1 |
|  | CC8-MRSA-IVa (PVL+), ACME-negative IS-88-like strain | 1 |
| CC8/ST72 (n=7) |  |  |
|  | ST72-MRSA-V/VT, WA MRSA-091 | 4 |
|  | ST72-MRSA-[V/VT+*fusC*] | 3 |
| CC9 (n=1) | ST834-MRSA-IV, WA MRSA-13 | 1 |
| CC15 (n=8) | CC15-MRSA-[V+*fusC*] | 8 |
| CC22 (n=82) |  |  |
|  | CC22-MRSA-IV (PVL+/*tst+*) | 35 |
|  | CC22-MRSA-IV (*tst1+*), "Gaza Epidemic Strain" | 24 |
|  | CC22-MRSA-IV (PVL+) | 19 |
|  | CC22-MRSA-[IV+*fusC+ccrAA/(C*)] | 1 |
|  | CC22-MRSA-IV (*fnbB-,sec/l+*), UK-EMRSA-15/Barnim EMRSA | 1 |
|  | CC22-MRSA-IV *(fnbB+*), related to UK-EMRSA-15/Barnim EMRSA | 1 |
|  | CC22-MRSA-IVc (PVL+) | 1 |
| CC30 (n=91) |  |  |
|  | CC30-MRSA-IV (PVL+), Southwest Pacific Clone | 68 |
|  | CC30-MRSA-[VI+*fusC*] (PVL+) | 13 |
|  | CC30-MRSA-IV (PVL-/*tst1-*) | 4 |
|  | CC30-MRSA-IV (PVL-/*tst1+*) | 3 |
|  | CC30-MRSA-V/VT (PVL+), WA MRSA-124 | 2 |
|  | CC30-MRSA-V/VT | 1 |
| CC45 (n=3) |  |  |
|  | CC45-MRSA-IVa, Berlin EMRSA | 1 |
|  | CC45-MRSA-[IV+*fusC+tir*] | 1 |
|  | CC45-MRSA-[VI+*fusC*] | 1 |
| CC59 (n=5) |  |  |
|  | CC59-MRSA-VT (*ermB+/aphA3+/*PVL+), Taiwan Clone | 4 |
|  | CC59-MRSA-VT (*ermB-/aphA3*-/PVL+) | 1 |
| CC80 (n=27) |  |  |
|  | CC80-MRSA-IV (PVL+) | 21^##^ |
|  | CC80-MRSA-IV (PVL-) | 3^#^ |
|  | CC80-MRSA-IVc (PVL+) | 2 |
|  | CC80-MRSA-IVc (PVL-) | 1 |
| CC88 (n=19) |  |  |
|  | CC88-MRSA-IV (PVL+) | 9 |
|  | CC88-MRSA-IV | 3 |
|  | CC88-MRSA-IV, WA MRSA-2 | 3 |
|  | CC88-MRSA-V (PVL+), WA MRSA-117 | 2 |
|  | CC88-MRSA-IV (*etA+*) | 1 |
|  | CC88-MRSA-VI | 1 |
|  |  |  |
| CC96 (n=1) | CC96-MRSA-IV | 1 |
| CC97 (n=22) |  |  |
|  | CC97-MRSA-[V/VT+*fusC*] | 13 |
|  | CC97-MRSA-[V+*fusC*] | 2 |
|  | CC97-MRSA-IV, WA MRSA-54/63 | 3 |
|  | CC97-MRSA-V/VT | 4 |
| CC121 (n=8) | CC121-MRSA-[V/VT+*fusC*] (PVL+) | 6 |
|  | CC121-MRSA-V/VT (PVL+) | 1 |
|  | CC121-MRSA-V/VT | 1 |
| CC152 (n=7) |  |  |
|  | CC152-MRSA-[V/VT+*fusC*] (PVL+) | 4^$^ |
|  | CC152-MRSA-V/VT (PVL+) | 2 |
|  | CC152-MRSA-IV (PVL+) | 1 |
| CC239 (n=11) |  |  |
|  | CC239-MRSA-[III+*ccrC*] | 3 |
|  | CC239-MRSA-[III+ccrC] | 3 |
|  | CC239-MRSA-[III+Cd/Hg+*ccrC*] | 3 |
|  | CC239-MRSA-[III+SCC*mer+ccrC*] | 2 |
| CC361 (n=35) |  |  |
|  | CC361-MRSA-[V/VT+*fusC*] | 20 |
|  | CC361-MRSA-IV, WA MRSA-29 | 7 |
|  | CC361-MRSA-V, WA MRSA-70 | 5 |
|  | CC361-MRSA-V/VT, WA MRSA-70/110 | 2 |
|  | CC361-MRSA-V | 1 |
| CC398 (n=3) |  |  |
|  | CC398-MRSA-V/VT (PVL+) | 2 |
|  | CC398-MRSA-VT (PVL+) | 1 |
| CC772 (n=36) |  |  |
|  | CC772-MRSA-V (PVL+), "Bengal Bay Clone" | 27 |
|  | CC772-MRSA-[V+kdp] (PVL+) | 2 |
|  | CC772-MRSA-VT (PVL+) | 4 |
|  | CC772-MRSA-V/VT (PVL+) | 3^##^ |
| CC1153 (n=4) |  |  |
|  | CC1153-MRSA-[V/VT+*fusC*] (PVL+) | 2 |
|  | CC1153-MRSA-[I+f*usC*] (PVL+) | 2 |
| CC2250 S. argenteus (n=1) ^###^ | CC2250-MRSA-IV, WA MRSA-114 | 1 |

*one strain harboured tirS as additional payload on SCC*mec;* ***two strains had SCCmec* VT ;  *****two strains harboured tirS as additional payload on SCC*mec; ^@^*two strains had SCC*mec* IVa ; ^#^one strain had SCC*mec* VT (GR1); ^##^SCC*mec* not subtyped; ^$^one strain had SCC*mec* V+*fusc*; ^###^Phenotypically identified as MRSA but found to be CC2250 *S. argenteus* on genotyping.
